# Editorial: Novel therapeutic targets of gastric carcinogenesis: from basic research to drug development and clinical application

**DOI:** 10.3389/fonc.2024.1431520

**Published:** 2024-05-23

**Authors:** Chenchen Niu, Dong Ren, Bella Lingjia Liu

**Affiliations:** ^1^ Department of Pathology and Laboratory Medicine, University of California, Irvine, Irvine, CA, United States; ^2^ Department of Pathology, Molecular and Cell-Based Medicine, Icahn School of Medicine at Mount Sinai, New York, NY, United States

**Keywords:** gastric cancer, carcinogenesis, therapeutic targets, drug development, clinical application

## Introduction

In 2020, gastric cancer (GC) was reported as the 5^th^ most common cancer and the 4^th^ leading cause of cancer-related death worldwide (1). The survival rate of GC patients remains low despite the progress of cancer treatments (2). Current therapeutic strategies which have proven efficacy for GC include systemic chemotherapy, radiotherapy, surgery, immunotherapy, and target therapy (3). Micro-satellite instability (MSI), programmed cell death ligand 1 (PD-L1), human epidermal growth factor receptor 2 (HER2), tumor mutation burden, and Epstein-Barr virus are useful biomarkers for selecting patients for immunotherapy and targeted therapy (3). Drugs of targeted therapy have been used for treating different molecular subtypes of GC including trastuzumab targeting HER2, ramucirumab targeting vascular endothelial growth factor receptor 2 (VEGFR2) (4), cetuximab targeting epidermal growth factor receptor (EGFR) (5), dovitinib and AZD4547 targeting fibroblast growth factor receptor 2 (FGFR2) (6).

A better understanding of the pathogenesis of GC at molecular level will help identify additional novel therapeutic targets for drug development and optimize the existing treatment to become more effective. The aim of the proposed Research Topic “*Novel therapeutic targets of gastric carcinogenesis: from basic research to drug development and clinical application*” is to provide updates and new findings regarding the pathogenesis and therapeutic strategies for GC.

## Overview of the articles included in this Research Topic

Runt-associated transcription factor 2 (RUNX2) plays an essential role during the osteoblast differentiation and bone development and distributes widely in different tissues (7). It has been reported that RUNX2 is closely related to the proliferation, invasion, and bone metastasis of multiple malignancies including osteosarcoma, breast cancer, prostate cancer, lung cancer, colon cancer, and GC (8). Several mechanisms have been studied to reveal how RUNX2 promotes the progression of GC including up-regulating the chemokine receptor CXCR4 (9), enhancing NID1 signaling (10), regulating COL1A1 (11), YAP1 (12) and FN1 expression (13). MGAT5, also known as N-acetylglucosamino transferase V, can remodel the tumor microenvironment and accelerate tumor cell growth and metastasis (14, 15). MMP13 has been reported to be activated in GC and as a downstream target of RUNX2 in breast cancer (16, 17). Wang et al. revealed a significant positive correlation between MGAT5, MMP13, and RUNX2 expression through the investigation of the GC samples in the database. They used JASPAR analysis to find one potential binding site of RUNX2 in the promoter regions of MGAT5 and MMP13 and demonstrated that RUNX2 could regulate the expression of MGAT5 and MMP13 respectively in the human GC cell lines. In addition, *in vivo* assay demonstrated that RUNX2 silencing could decrease the tumor size and cancer metastasis through decreasing the expression levels of MGAT5 and MMP13.

Signal transducer and activator of transcription (STAT) 3 is currently considered an oncogene and plays a crucial role in tumorigenesis (18) which makes STAT3 an attractive therapeutic target for cancer therapy. Liu et al. used structure-based virtual screening complemented by molecular dynamics simulations to identify ten small molecules with potential STAT3 inhibitory activity. The biological assays demonstrated that the three of the ten compounds (compounds 4, 7, and 10) could suppress the proliferation of three types of GC cell lines. Among the three candidates compound 4 had the most potent efficacy. It could displace the specific STAT3-SH2 binding peptide *in vitro*, had the dose-dependent inhibitory action on the phosphorylation of STAT3 at Tyr-705 triggered by IL-6, and was able to disrupt the mitochondrial oxidative phosphorylation (OXPHOS) pathways. The group will continue working on the structural optimization of compound 4 for developing a novel STAT3 inhibitor for the treatment of GC.

Systemic chemotherapy is the standard first line therapy for patients with HER2 negative advanced gastric cancer (AGC) (19). Because the overall survival time of most patients with AGC is within 12 months of diagnosis, it is important to develop a predictive model to evaluate the treatment’s efficacy and prognosis which can help optimize the treatment plan (20). Yang et al. did a prospective study to investigate the predictive value of the clinicopathologic features together with laboratory hematologic indicators including multiple immune-inflammatory biomarkers such as serum vascular endothelial growth factor A (VEGFA), systemic immune-inflammation index (SII), and c-reactive protein/albumin ratio (CAR) in HER2-negative AGC. They established an immune-inflammation-based predictive model for OS, and for the effectiveness of the first-line chemotherapy and the progression-free survival (PFS) of patients. The model was integrated with the indicators in the blood and became more clinical applicable. It provided a novel approach for screening the primary chemotherapy-resistant populations.

Approximately 17–20% of patients with GC have the HER2 protein overexpression, especially in intestinal-type gastric cancer and cancers of the proximal stomach or the gastroesophageal junction (21). Anti-HER2 antibody trastuzumab is the only molecular targeted drug approved by US Food and Drug Administration (FDA) for the first-line treatment of HER2 positive AGC (22). However, the high rate of resistance limits its clinical benefits (22). The mechanism of trastuzumab resistance in GC is not well understood. The disruption of circadian rhythm can induce or increase the risk of cancer by altering tumor suppressors and oncogenes (23). Wang et al. did an extensive and detailed review on the studies related to how circadian clock regulating the progression of cancer, circadian clock as a drug target in cancer therapy, the metabolic rhythm reprogramming as a possible mechanism accounting for trastuzumab resistance. The study published in Cancer Research in 2022 demonstrated trastuzumab-resistant GC cells had high glycolytic activity controlled by hexokinase 2 (HK2)-dependent glycolysis with a circadian pattern and proved a metformin-based chronotherapy could overcome trastuzumab resistance in HER2 positive AGC (22).

## Conclusion

In summary, articles in this Research Topic provided the progress of basic research for the novel mechanism of the pathogenesis of GC to identify potential therapeutic targets, the mechanism for the trastuzumab resistance in HER2 positive GC which could be overcome by metformin-based chronotherapy, and an immune-inflammation-based predictive model for treatment effectiveness and prognosis. Further understanding the pathogenesis of GC is necessary and will potentially lead to the development of personalized prevention and treatment approaches to benefit the patients.

## Author contributions

CN: Writing – review & editing, Writing – original draft, Supervision, Conceptualization. DR: Writing – review & editing, Writing – original draft, Supervision, Conceptualization. BL: Writing – review & editing, Writing – original draft, Supervision, Conceptualization.
